# Breakfast Cereals Carrying Fibre-Related Claims: Do They Have a Better Nutritional Composition Than Those without Such Claims? Results from the Food Labelling of Italian Products (FLIP) Study

**DOI:** 10.3390/foods10092225

**Published:** 2021-09-19

**Authors:** Daniela Martini, Cristian Del Bo’, Mauro Serafini, Marisa Porrini, Nicoletta Pellegrini, Donato Angelino, on behalf of SINU Young Working Group

**Affiliations:** 1 Department of Food, Environmental and Nutritional Sciences (DeFENS), Università degli Studi di Milano, 20133 Milan, Italy; daniela.martini@unimi.it (D.M.); cristian.delbo@unimi.it (C.D.B.); marisa.porrini@unimi.it (M.P.); 2 Faculty of Bioscience and Technology for Food, Agriculture and Environment, University of Teramo, 64100 Teramo, Italy; mserafini@unite.it (M.S.); dangelino@unite.it (D.A.); 3 Department of Agricultural, Food, Environmental and Animal Sciences, University of Udine, 33100 Udine, Italy

**Keywords:** fibre, nutrition claim, nutritional declaration, nutritional quality, food labelling

## Abstract

Nutrition claims (NCs) on food packaging are, from one side, an informative tool about the nutritional characteristics of the product. From another side, they could bias the consumer in perceiving such products as healthier than those without claims. In order to investigate whether products with NCs have a better nutritional composition that those without claims, the present study aimed to compare the nutrition facts of 376 breakfast cereals, with and without fibre-related NCs, available in 13 Italian retailer online stores. Among these products, 73 items claimed to be a “source of fibre” and 109 “high in fibre”. In addition to a higher fibre content, products “high in fibre” showed higher protein and fat contents but lower carbohydrate, sugar and salt contents compared to both “source of fibre” and without fibre-related NC items. Overall, a high variability in nutritional values was also observed within products with the same type of fibre-related NC. In conclusion, the results suggested that fibre-related NCs should not be considered as a marker of a better nutritional composition of breakfast cereals, and thus consumers should be educated to carefully read the entire nutritional information reported in the food labelling.

## 1. Introduction

Dietary fibre is defined by the European Food Safety Authority as “non-digestible carbohydrates plus lignin, including non-starch polysaccharides—cellulose, hemicelluloses, pectins, hydrocolloids (i.e., gums, mucilages, glucans), resistant oligosaccharides, fructo-oligosaccharides, galactooligosaccharides, other resistant oligosaccharides, resistant starch—consisting of physically enclosed starch, some types of raw starch granules, retrograded amylose, chemically and/or physically modified starches, and lignin associated with the dietary fibre polysaccharides” [1].

A large body of evidence suggests that an adequate intake of fibre is inversely associated with the risk of many non-communicable diseases, including intestinal bowel disease [2] and some types of cancer [3,4]. Dietary fibre is one of the main bioactive components of plant-based foods, with whole grain cereals, pulses, fruits and vegetables as the main sources [5]. In Italy, a daily reference intake of 12.6–16.7 g fibre/1000 kcal is recommended (8.4 g/1000 kcal during childhood), with a suggested dietary target of at least 25 g/day for the prevention of chronic diseases [6]. However, the population intake is still far from the reference amount, as shown in the latest available survey of the Italian nutrient intakes (INRAN-SCAI survey, [7]).

Based on the Regulation (EU) n. 1169/2011 [8], in Europe the indication of fibre content in the nutrition declaration is not mandatory, but it can be stated on a voluntary basis. Despite this, as stated by the Regulation (CE) n. 1924/2006 [9], when a nutrition claim (NC) and/or health claim (HC) is made for a nutrient, the amount of that nutrient must be declared. This Regulation declares that two different claims specific to fibre can be used: “source of fibre” for products containing at least 3 g of fibre per 100 g or at least 1.5 g of fibre per 100 kcal, and “high in fibre”, for 6 g of fibre per 100 g or at least 3 g of fibre per 100 kcal [9].

The presence of NCs on the packaging should be of help to consumers in making informed food choices by giving information on the nutrition characteristics of the product. However, it has been suggested that the presence of NCs on the food packaging, highlighting certain “positive characteristics” of the food, can lead consumers to perceive those foods as healthier than products not indicating claims, thus creating the so-called “halo effect” [10,11]. This may even lead to the consumption of an inappropriate portion size of the food, affecting the consumption behaviour and possibly, in turn, the health status [12,13]. This aspect is particularly important considering that evidence shows that attention to, and use of, NCs is not very widespread among Italian consumers and that the level of understanding of the selected claims is quite low, with high risk of misinterpretation and confusion [14]. Thus, a nutritional comparison between products with and without NC is worthy of investigation, to demonstrate whether the former have a better nutrition composition than the latter. Thus far, few studies have been conducted with this purpose to investigate the impact of different types of NC on the nutritional composition of several categories of food products [15,16,17], but evidence about the presence of a fibre-related NC as a marker of the overall nutritional quality of food products is still inconclusive.

Based on these considerations, the aims of the present study were to: (1) evaluate the nutritional composition of breakfast cereals currently sold in Italy by retrieving information from the food packaging and (2) investigate differences in terms of energy, macronutrients and salt contents among products with and without fibre-related NCs. These products were chosen since they bear a high number of NCs [18]. This work was conceived within the Food Labelling of Italian Products (FLIP) study which systematically investigates the nutritional quality of food products currently sold in Italy.

## 2. Materials and Methods

### 2.1. Food Product Selection

The home-shopping websites of the major retailers present on the Italian market were used for the selection of breakfast cereals, as previously reported [19]. The online search was conducted in 2018 and updated in March 2021. All breakfast cereals sold in at least one website were considered eligible for inclusion into the dataset. The exclusion criteria for product selection were: (i) products that were not prepacked; (ii) not all sides of the packaging available; (iii) nutrition declaration or list of ingredients not clear in the images; (iv) unavailability of products in all the online stores during the entire data collection period.

### 2.2. Data Extraction

For each included food item, the following information was retrieved from the images of the packaging and collected in a dataset: company name, brand name, descriptive name, energy (kcal/100 g), total fat (g/100 g), saturates (g/100 g), total carbohydrates (g/100 g), sugars (g/100 g), protein (g/100 g), salt (g/100 g) and fibre (g/100 g). Furthermore, additional information, such as presence and type of NC or HC, gluten free (GF) declaration (presence or absence of gluten), organic declaration (presence or absence of organic declaration) and brand (branded or private label product) was collected. Regarding NCs, claims related to fats, sugars, protein, salt, vitamins and minerals were searched for, considering both absolute and comparative claims and NCs having the same meaning for consumers as stated by the Regulation 1924/2006. Specifically, for NCs related to fibre, the presence of claims “source of fibre” and “high in fibre” was distinguished. In detail, “with fibre” was considered equivalent to “source of fibre” while “rich in fibre” was considered equivalent to “high in fibre”, as implemented in previous work [20]. Only NC displayed as text were considered while NCs shown as pictorial or symbolic representations were excluded.

Two researchers (DM and DA) were responsible for the accuracy of the retrieved information and disagreements were discussed and solved with the consultation of a third researcher (CDB). To compare items, they were subgrouped by considering (i) type and (ii) presence/absence of fibre-related NCs. Based on the descriptive name, breakfast cereals were classified in 6 types: cereal bars, muesli, flakes, bran cereals, puffed cereals and others (e.g., cereals with honey, cream-filled cereals). Definitions and examples of types and categories were provided in a previous publication [19].

### 2.3. Statistical Analysis

The IBM SPSS statistics for Macintosh Version 27.0 (IBM Corp., Armonk, NY, USA) was used to perform the statistical analysis, setting the significant level at *p* < 0.05. The Kolmogorov–Smirnov test was used to verify the normality of data distribution that was rejected. Variables were expressed as median and (interquartile range). Data of energy, macronutrient, salt and fibre contents per 100 g of product, with interquartile ranges stated in parentheses, were analysed using (i) the Kruskal–Wallis non-parametric one-way ANOVA for independent samples with multiple pairwise comparisons for differences among fibre-related NC categories; (ii) within the three categories of products (high in fibre, source of fibre and without claim on fibre), the Mann–Whitney non-parametric test for two independent samples was applied for differences between variables of products with or without other declarations. Finally, a Principal Component analysis (PCA) with varimax rotation was performed for all products, considering energy, macronutrient, salt and fibre contents per 100 g of product, to better elucidate the inter-product nutritional variability.

## 3. Results

### 3.1. Characteristics of the Considered Products

The number and the types of items retrieved from the online stores are reported in Table 1. For all the 376 breakfast cereals included in the present analysis, the fibre content was present in the nutrition declaration and in 336 items it was ≥3 g/100 g that would allow them to bear a fibre-related NC. Among the 267 items reporting at least one NC, 182 (~48% of the total) reported a fibre-related NC (source of fibre: N = 73; high in fibre: N = 109), while no comparative claims were found. A 100% compliance rate was found, with no items reporting incorrect information. The rate of products indicating one of the two NCs was the highest for bran cereals (86% out of the total), followed by cereal bars (58%) and muesli (55%). Lower numbers of NC were observed in flakes (45%), puffed cereals (21%) and other cereals (44%). Overall, fibre-related NCs were the most frequent ones, followed by NC on vitamins and minerals (N = 135), fat (N = 42), sugars (N = 24) and salt (N = 16). Specifically for the 182 items bearing a fibre-related NC, 63 also carried a NC on vitamins and minerals, 21 on fat, 17 on sugar, 15 on salt and 7 on protein. Fibre claims prevailed on branded products compared to those sold by private labels (N = 107 vs. 75), and in conventional items compared to the organic ones (N = 138 vs. 44). Finally, a similar percentage of products carrying claims related to fibre, i.e., 50 and 48%, were found in GF and gluten-containing products, respectively, despite the large difference in terms of numbers of items.

### 3.2. Nutritional Composition of Products with and without Fibre-Related Nutrition Claims

The nutritional quality of breakfast cereals with and without fibre-related NC is reported in Table 2. In addition to the expected higher fibre content, products claiming to be “high in fibre” showed higher fat and protein contents but a lower carbohydrate, sugar and salt content compared to “source of fibre” and products without a fibre-related NC. Conversely, energy content in products without a fibre-related NC was higher compared to “high in fibre” but not “source of fibre” products, while saturates content did not differ among categories. It is worth highlighting that also the products without a fibre-related NC had a quite remarkable fibre content (median value of 4.5 g/100 g, compared to 5.2 and 9.0 g/100 g in “source of fibre” and “high in fibre” products, respectively). A complete overview of the nutritional composition of the breakfast cereals organised by types of products, and which considers the absence or presence of the fibre-related NC, is listed in Supplementary Table S1. As previously mentioned, most food types (i.e., cereal bars, flakes, puffed cereals, other cereals), with the only exception of muesli and bran cereals, claiming to be “high in fibre” showed higher fibre content compared to products not bearing fibre-related NC. Among these, cereal bars, flakes and other cereals with a “high in fibre” NC were also lower in total carbohydrates compared to other products, as well as higher in protein and total fat, despite only flakes were higher in saturates; sugars in cereal bars and other cereals “high in fibre” were higher than in products without NCs, but not in the ones claiming to be a “source of fibre”. Bran cereals with or without a fibre-related NC were the only types of product that did not differ in any of the nutrients or in their energy contents. The whole dataset of the products organised based on presence or absence or fibre-related claims and then for the presence or absence of other NCs, HC, organic and GF declaration and brand can be found in Supplementary Table S2. Once again, very few significant differences emerged from the comparison of the products based on the other NCs, mainly for sugar contents.

**Figure 1:**
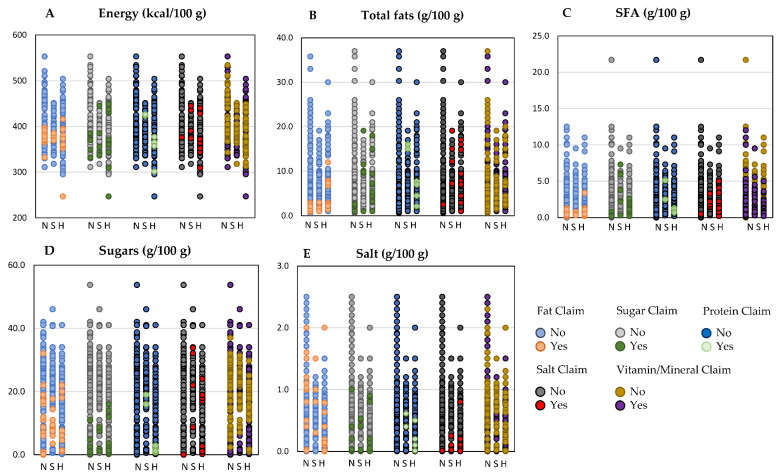
Energy (**A**); nutrients (**B**–**D**) and salt (**E**) of the considered breakfast cereal products, carrying or not fibre-related and other nutrition claims. Legend: N, no fibre-claim; S, source of fibre; H, high in fibre; SFA, saturates.

Figure 1 shows the variability in terms of energy (Figure 1A), nutrients (Figure 1B–D) and salt (Figure 1E) content among the 376 items without fibre-related NC (N), or with “source of fibre” (S) and “high in fibre” (H) NCs, further stratified considering the coexistence or not of other NCs, i.e., fat, sugar, protein, salt and vitamin/mineral claims.

As mentioned above, nutritional characteristics showed a high variability within categories of products differing for the presence or absence of fibre-related claims. Energy, nutrient and salt content seemed to be affected by the specific type of NC. For instance, the lowest fat, sugar and salt contents were observed in products with fat, sugar, and salt NCs, regardless of the presence of fibre-related claims (Figure 1B,D,E).

**Figure 2:**
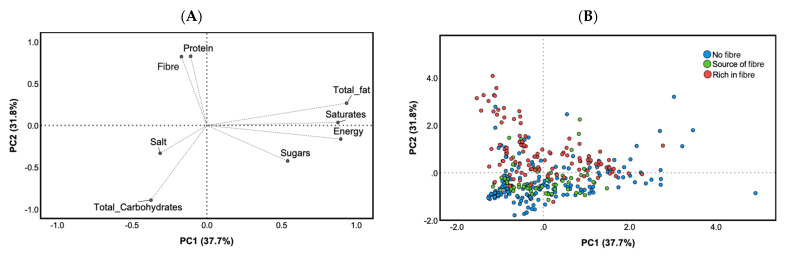
Principal component analysis (PCA) describing the intra-category variability of products based on their nutrient composition (energy (kcal/100 g), total fat (g/100 g), saturates (g/100 g), total carbohydrates (g/100 g), sugars (g/100 g), protein (g/100 g), fibre (g/100 g) and salt (g/100 g)). Loading plots of Principal Component (PC) 1 and 2 (**A**) and score plots of the nutrient composition of each product analysed organised according to presence and kind of fibre-related claim from PC1 and PC2 (**B**).

The variability in the nutritional composition of the breakfast cereals was explored by PCA (Figure 2). Two PCs described a total of 69% of the total variability. The PC1, describing the 37.7% of the variability, was positively loaded by energy, total fat, saturates and sugars, and negatively loaded by protein, fibre, salt and total carbohydrates. PC2, describing the 31.8% of the variability, was positively loaded by protein and fibre and negatively by salt and total carbohydrates (Figure 2A). A high inter-product variability was observed for the three categories of breakfast cereals (“high in fibre”, “source of fibre” or without fibre-related NCs), regardless the presence of claims on fibre. On the whole, most of the products were described by a high content of total carbohydrates, salt (negatively loaded by both PCs) and only some “rich in fibre” products were distributed positively on PC2 (Figure 2B).

## 4. Discussion

To the best of our knowledge, this is the first survey evaluating the nutritional composition of almost four hundred breakfast cereals sold on the Italian market by focusing on the presence or absence of fibre-related NCs. Briefly, this survey revealed that ~48% of items carried a fibre-related NC and that products claiming to be “high in fibre” showed higher protein and fat contents but lower total carbohydrate, sugar and salt contents compared to both “source of fibre” and without fibre-related NC items. On the whole, a high variability in terms of nutritional values was observed among items.

In detail, the first finding worthy of consideration is that, although non-mandatory, all the retrieved items reported the fibre content in the nutrition declaration. Moreover, although about 90% of the products contain more than 3 g/100 g of fibre, more than half of the items do not bear a fibre-related claim on the packaging. This aspect has been also considered in a Canadian survey where the nutritional quality of almost one thousand cereal and other grain products has been evaluated, and even 89% of the items not carrying a NC resulted to be eligible for it [21].

Another interesting finding is the presence of several types of NCs for breakfast cereal products, with most of the items bearing the fibre-related claim carrying also at least another claim, related to both “healthy” (i.e., vitamins and minerals) and “unhealthy” (i.e., fat, salt) substances, which are differently perceived by consumers and, then, differently ranked as preferred claims by consumers [22].

Intriguingly, no incorrect information was observed in any of the items, thus showing a 100% compliance with the Regulation 1924/2006. These results are higher than those reported in a previous investigation performed in Spain in which only 70.9% of NCs breakfast cereals and flakes were compliant [20], although fibre was the nutrient with the most correct NCs.

The prevalence of NCs in Europe largely varies across countries, food categories and type of NC. This last aspect was the focus of the EU funded project CLYMBOL (“Role of health-related CLaims and sYMBOLs in consumer behaviour”) in which, among 2034 foods and drinks, 20.8% carried at least one NC, with a range from 16.0% in Germany to 29.6% in the UK, and most of the NCs were related to vitamins, fats and sugars [23]. Although the survey did not consider the Italian market, authors reported that at least one third of the prepacked products belonging to “cereals and cereal products” carried a NC [23]. Similar findings have been retrieved from a recent Spanish survey where, among the 3197 surveyed foods, the 36.1% carried NCs, at a rate of 3.3 NCs/food, with nuts and seeds, legumes and non-alcoholic beverages reporting the highest prevalence and NCs on micronutrients, fat, fibre and sugars as the most common [20]. Once again, the “cereal” category, with more than 40% of the products carrying a NC, was the most representative category for number of NC [20]. A recent paper of Prada et al. concerning 289 breakfast cereals sold in Portugal highlighted similar results, with an average of 3.9 total claims per product, 70% of which related to the nutrient composition (no data on fibre-related claim) [18]. Similarly, Davidovic et al. [24] recently observed that 81.3% of breakfast cereals sold on the Serbian market displayed at least one NC.

As stated by the Regulation (EC) No 1924/2006 [9], NCs in Europe are regulated with the aim to ensure a high level of protection for consumers and to facilitate their choice. From one side, several studies performed so far highlighted that choosing products with NCs may lead to an improvement of the total quality of the diet [25]. However, from another side, it has also been observed that NCs may influence consumers, in particular the “restrained eaters” through the so-called “health halo” effect, which leads people to perceive a food as “healthy” only because of carrying nutrition or health claims on the packaging [26]. This perceived healthiness may lead to the consumption of larger portion sizes, overeating and thus to an overall increase in energy intake [27,28]. A recent Chilean survey on breakfast cereals pointed out that not only products carrying NCs are perceived as healthier than the regular ones, but among the NCs, the fibre-related claims were the main driver of this perceived healthiness [29]. Moreover, the presence of NC on fibre produced the “health halo” effect by influencing consumers’ perceptions of the overall healthiness, vitamin content, naturalness and quality of the product [29].

For these reasons, it is of interest to investigate if this consumer perception corresponds to real differences in the nutritional quality of products with or without NC.

Despite some statistically significant differences, from a nutritional point of view, we found only small differences in terms of lower energy, sugar and salt in products carrying these NCs compared to those that did not. In particular, the nutritional quality of products claiming to be “high in fibre” was different from the other products in several aspects; on the contrary, only small differences were observed between products that were a “source of fibre” and products without a fibre-related NC, which is probably partially due to the fact that many products did not carry a fibre-related NC despite having more than 3 g fibre per 100 g. A comparison with previous findings is not easy since many studies performed so far focused on different types of products and NCs. More generally, some published studies just compared the nutritional quality of several products carrying no other NCs. Despite studies focused on different markets and different types of products [15,17,30], authors often concluded that only a small difference existed when comparing products with or without NCs, and these results may also depend on the type of NC. For instance, a recent survey on US fruit drinks showed that products with calorie- and sugar-related claims were effectively lower in calories and sugars than the products free of claims, while no differences were observed in products carrying other types of claims (e.g., related to vitamin C), which resulted higher in calories and sugars compared to products with no claims [16].

The present work has some strengths and limitations, mainly in relation to the collection methodology of the data. First of all, the number and the type of products retrieved on the online shops may result not exhaustive and representative of the products consumed by Italians, as more other products sold on local shops or discounts should be considered. However, also by referring to the above discussed surveys, one of the strengths of the present work is just the number of considered products, which is particularly high. Further, as previously stated, we found that among the products allowed to bear a fibre-related claim, a good amount chose not to indicate it on the packaging. This may represent a bias in reporting the real picture of the nutritional quality of the breakfast cereals carrying the fibre-related NC or not, in terms of energy, macronutrients and salt contents. However, the aim of the FLIP project was to use the only information displayed on the packaging and hence available to the consumer while shopping. Finally, some comparisons performed in the study were carried out among subcategories with small sample sizes (e.g., very few bran cereals did not report a fibre claim) and this may have affected the outcomes within these product categories.

## 5. Conclusions

In conclusion, we showed for the first time that the presence of a fibre-related nutritional claim indicated on the packaging of breakfast cereals sold in Italy should not be considered as a proxy of the general nutritional quality of the food items. Indeed, results from the present study suggest that the simple consideration of purchasing breakfast cereal products carrying a fibre-related NC does not guarantee the purchase of products with an improved nutritional quality compared to the regular ones. This is probably because several products did not report a fibre-related claim despite having a fibre content that would allow them to bear these types of claims. Moreover, the presence of additional ingredients other than cereals may lead to a very different nutritional composition of the whole product. Thus, nutritional education activities are needed to train the consumer in carefully reading the food labels, understanding the meaning of the values and information on the packaging and, in turn, making conscious purchases.

## Figures and Tables

**Table 1:** Descriptive analysis of the breakfast cereals nutritional data available through online stores in Italy.

		Number of Products	Without NC on Fibre (% of the Total)	With a Fibre-Related NC	
				Source of Fibre (% of the Total)	High in Fibre (% of the Total)
Total (N)		376	194 (52%)	73 (19%)	109 (29%)
Type	Cereal bars	57	24 (42%)	24 (42%)	9 (16%)
	Muesli	62	28 (45%)	5 (8%)	29 (47%)
	Flakes	132	72 (55%)	29 (22%)	31 (23%)
	Bran cereals	21	3 (14%)	0	18 (86%)
	Puffed cereals	38	30 (79%)	3 (8%)	5 (13%)
	Others	66	37 (56%)	12 (18%)	17 (26%)
NC on fat	No	334	173 (52%)	62 (19%)	99 (30%)
	Yes	42	21 (50%)	11 (26%)	10 (24%)
NC on sugar	No	352	187 (53%)	67 (19%)	98 (28%)
	Yes	24	7 (29%)	6 (26%)	11 (24%)
NC on protein	No	369	194 (53%)	71 (19%)	104 (28%)
	Yes	7	0	2 (29%)	5 (71%)
NC on salt	No	360	193 (54%)	69 (19%)	98 (28%)
	Yes	16	1 (6%)	4 (25%)	11 (69%)
NC on vitamins or minerals	No	241	122 (51%)	37 (15%)	82 (34%)
	Yes	135	72 (53%)	36 (27%)	27 (20%)
Health claim	No	306	170 (56%)	52 (17%)	84 (27%)
	Yes	70	24 (34%)	21 (30%)	25 (36%)
Gluten free	No	356	184 (52%)	66 (19%)	106 (30%)
	Yes	20	10 (50%)	7 (35%)	3 (15%)
Organic	No	277	139 (50%)	61 (22%)	77 (28%)
	Yes	99	55 (56%)	12 (12%)	32 (32%)
Branded	No	187	112 (60%)	36 (19%)	39 (21%)
	Yes	189	82 (43%)	37 (20%)	70 (37%)

Legend: N, number; NC, nutrition claim.

**Table 2:** Nutritional quality of breakfast cereals with and without fibre-related nutrition claims.

	Without Claim on Fibre	With Fibre-Related NC	
		Source of Fibre	High in Fibre
Energy (kcal/100 g)	385 (375–408) a	382 (375–394) ab	377 (360–431) b
Total fat (g/100 g)	3.8 (1.6–10.3) b	5.0 (2.3–9.2) b	7.3 (3.9–15.0) a
SFA (g/100 g)	1.1 (0.4–3.5) a	1.6 (0.5–3.5) a	1.3 (0.8–2.6) a
Total carbohydrates (g/100 g)	76.0 (66.0–81.4) a	75.1 (67.0–79.0) a	61.0 (56.0–65.0) b
Sugars (g/100 g)	21.0 (7.7–28.0) a	20.0 (11.0–26.0) a	17.0 (6.2–21.0) b
Fibre (g/100 g)	4.5 (3.0–6.5) c	5.2 (4.2–5.8) b	9.0 (7.5–14.0) a
Protein (g/100 g)	8.0 (7.0–9.3) b	7.5(6.7–8.9) b	10.1 (8.7–13.0) a
Salt (g/100 g)	0.6 (0.2–1.0) a	0.5 (0.3–0.8) a	0.3 (0.1–0.6) b

Legend: For each category, different letters in the same row after parenthesis indicate significant differences among types (Kruskal–Wallis test for independent samples with multiple pairwise comparisons, *p* < 0.05). Legend: NC, nutrition claim; SFA, saturates.

## Supplementary Materials

The following are available online at https://www.mdpi.com/article/10.3390/foods10092225/s1, Table S1: Energy, macronutrients and salt across breakfast cereal types organised according to the absence or presence of fibre-related nutrition claims; Table S2: Energy, macronutrients and salt content across breakfast cereals organised according to the absence or presence of a fibre-related nutrition claim by considering the presence or absence of other nutrition claims, health claims or other declarations on the food packaging.

## Appendix A

**SINU Young Working Group**

- Margherita Dall’Asta, Department of Animal Science, Food and Nutrition, Università Cattolica del Sacro Cuore, Piacenza, Italy
- Marika Dello Russo, Institute of Food Sciences, National Research Council, Avellino, Italy
- Daniele Nucci, Veneto Institute of Oncology IOV-IRCCS, Padova, Italy
- Stefania Moccia, Institute of Food Sciences, National Research Council, Avellino, Italy
- Gaetana Paolella, Department of Chemistry and Biology A. Zambelli, University of Salerno, Fisciano, Italy
- Veronica Pignone, Freelance Nutritionist, San Giorgio del Sannio, Italy
- Alice Rosi, Department of Food and Drugs, University of Parma, Parma, Italy
- Emilia Ruggiero, Department of Epidemiology and Prevention, IRCCS Neuromed, Pozzilli, Italy
- Carmela Spagnuolo, Institute of Food Sciences, National Research Council, Avellino, Italy
- Giorgia Vici, School of Biosciences and Veterinary Medicine, University of Camerino, Camerino, Italy

## References

[1] EFSA Panel on Dietetic Products, Nutrition, and Allergies (NDA) (2010). Scientific opinion on dietary reference values for carbohydrates and dietary fibre. EFSA J..

[2] Milajerdi A., Ebrahimi-Daryani N., Dieleman L.A., Larijani B., Esmaillzadeh A. (2021). Association of dietary fiber, fruit, and vegetable consumption with risk of inflammatory bowel disease: A systematic review and meta-analysis. Adv. Nutr..

[3] Gianfredi V., Nucci D., Salvatori T., Dallagiacoma G., Fatigoni C., Moretti M., Realdon S. (2019). Rectal cancer: 20% risk reduction thanks to dietary fibre intake. systematic review and meta-analysis. Nutrients.

[4] World Cancer Research Fund International (2018). Diet, Nutrition, Physical Activity and Cancer: A Global Perspective—The Third Expert Report.

[5] Dhingra D., Michael M., Rajput H., Patil R.T. (2012). Dietary fibre in foods: A review. J. Food Sci. Technol..

[6] Italian Society of Human Nutrition (SINU) (2014). Livelli di Assunzione di Riferimento di Nutrienti ed Energia per la Popolazione Italiana.

[7] Sette S., Le Donne C., Piccinelli R., Mistura L., Ferrari M., Leclercq C., Arcella D., Bevilacqua N., Buonocore P., Capriotti M. (2013). The third National Food Consumption Survey, INRAN-SCAI 2005-06: Major dietary sources of nutrients in Italy. Int. J. Food Sci. Nutr..

[8] European Union Council (2011). Regulation No 1169/2011 on the provision of food information to consumers. Off. J. Eur. Union.

[9] European Union Council (2006). Regulation No 1924/2006 on nutrition and health claims made on foods. Off. J. Eur. Union.

[10] Chandon P. (2013). How package design and packaged-based marketing claims lead to overeating. Appl. Econ. Perspect. Policy.

[11] Fernan C., Schuldt J.P., Niederdeppe J. (2018). Health halo effects from product titles and nutrient content claims in the context of “protein” bars. Health Commun..

[12] Faulkner G.P., Pourshahidi L.K., Wallace J.M.W., Kerr M.A., McCaffrey T.A., Livingstone M.B.E. (2014). Perceived “healthiness” of foods can influence consumers’ estimations of energy density and appropriate portion size. Int. J. Obes..

[13] Benson T., Lavelle F., Bucher T., McCloat A., Mooney E., Egan B., Collins C.E., Dean M. (2018). The impact of nutrition and health claims on consumer perceptions and portion size selection: Results from a nationally representative survey. Nutrients.

[14] Annunziata A., Mariani A. (2019). Do Consumers Care about Nutrition and Health Claims? Some Evidence from Italy. Nutrients.

[15] Kaur A., Scarborough P., Matthews A., Payne S., Mizdrak A., Rayner M. (2016). How many foods in the UK carry health and nutrition claims, and are they healthier than those that do not?. Public Health Nutr..

[16] Duffy E.W., Hall M.G., Dillman Carpentier F.R., Musicus A.A., Meyer M.L., Rimm E., Smith Taillie L. (2021). Nutrition claims on fruit drinks are inconsistent indicators of nutritional profile: A content analysis of fruit drinks purchased by households with young children. J. Acad. Nutr. Diet..

[17] Taillie L.S., Ng S.W., Xue Y., Busey E., Harding M. (2017). No fat, no sugar, no salt… No problem? Prevalence of “low-Content” nutrient claims and their associations with the nutritional profile of food and beverage purchases in the United States. J. Acad. Nutr. Diet..

[18] Prada M., Saraiva M., Viegas C., Cavalheiro B.P., Garrido M.V. (2021). Examining the relationship between sugar content, packaging features, and food claims of breakfast cereals. Nutrients.

[19] Angelino D., Rosi A., Dall’Asta M., Pellegrini N., Martini D. (2019). Evaluation of the nutritional quality of breakfast cereals sold on the Italian market: The Food Labelling of Italian Products (FLIP) study. Nutrients.

[20] Ropero A.B., Blain N., Beltrá M. (2020). Nutrition claims frequency and compliance in a food sample of the Spanish market: The BADALI Study. Nutrients.

[21] Franco-Arellano B., Labonté M.-È., Bernstein J., L’Abbé M. (2018). Examining the nutritional quality of Canadian packaged foods and beverages with and without nutrition claims. Nutrients.

[22] (2019). Gracia; Barreiro-Hurlé Making Sense of Information Overload: Consumer Ranking of Nutritional Claims in Cereal Based Products. Nutrients.

[23] Hieke S., Kuljanic N., Pravst I., Miklavec K., Kaur A., Brown K.A., Egan B.M., Pfeifer K., Gracia A., Rayner M. (2016). Prevalence of nutrition and health-related claimson pre-packaged foods: A five-country study in europe. Nutrients.

[24] Davidović D., Paunović K., Zarić D., Jovanović A., Vasiljević N., Stošović D., Tomanić M. (2021). Nutrition and Health Claims Spectra of Pre-Packaged Foods on Serbian Supermarket Shelves: A Repeated Cross-Sectional Study. Nutrients.

[25] Williams P. (2005). Consumer understanding and use of health claims for foods. Nutr. Rev..

[26] Provencher V., Jacob R. (2016). Impact of perceived healthiness of food on food choices and intake. Curr. Obes. Rep..

[27] Oostenbach L.H., Slits E., Robinson E., Sacks G. (2019). Systematic review of the impact of nutrition claims related to fat, sugar and energy content on food choices and energy intake. BMC Public Health.

[28] Brown H.M., Rollo M.E., De Vlieger N.M., Collins C.E., Bucher T. (2018). Influence of the nutrition and health information presented on food labels on portion size consumed: A systematic review. Nutr. Rev..

[29] Mediano Stoltze F., Busey E., Taillie L.S., Dillman Carpentier F.R. (2021). Impact of warning labels on reducing health halo effects of nutrient content claims on breakfast cereal packages: A mixed-measures experiment. Appetite.

[30] Kaur A., Scarborough P., Hieke S., Kusar A., Pravst I., Raats M., Rayner M. (2016). The nutritional quality of foods carrying health-related claims in Germany, the Netherlands, Spain, Slovenia and the United Kingdom. Eur. J. Clin. Nutr..

