# Supplementary material for: Breakfast Cereals Carrying Fibre-Related Claims: Do They Have a Better Nutritional Composition Than Those without Such Claims? Results from the Food Labelling of Italian Products (FLIP) Study
Source: Foods. 2021 Sep 19;10(9):2225. doi: 10.3390/foods10092225 (PMC8467444; doi:10.3390/foods10092225)
Supplement: Supplementary file 1 [file foods-10-02225-s001.zip › Suppl Table 1.pdf]

Supplementary Table S1. Energy, macronutrients, and salt content across breakfast cereal types organised according to the absence or presence of fibre-related nutrition claims.

		Energy (kcal/100 g)	Total fat (g/100 g)	SFA (g/100 g)	Total carbohydrates (g/100 g)	Sugars (g/100 g)	Fibre (g/100 g)	Protein (g/100 g)	Salt (g/100 g)
**Cereal bars**	**NO**	401 (388–468) a	9.8 (8.2–20.0) a,b	4.4 (2.4–5.2)	67.5 (56.0–70.7) a	27.3 (25.0–30.0) a	4.8 (3.5–6.6) b	7.2 (6.2–10.5) a	0.6 (0.3–0.7)
	**SF**	384 (374–402) b	9.4 (7.3–12.0) b	3.9 (3.0–5.8)	67.6 (65.0–70.4) a	26.2 (17.5–27.7) a,b	5.5 (4.9–5.9) b	6.3 (5.6–7.0) b	0.5 (0.4–0.7)
	**HF**	419 (411–442) a	17.0 (13.5–20.0) a	3.5 (2.3–4.3)	55.5 (49.0–56.5) b	19.0 (17.5–22.0) b	12.5 (7.9–14.0) a	9.0 (8.0–9.7) a	0.3 (0.3–0.4)
**Muesli**	**NO**	402 (388–469)	10.0 (8.2–20.0)	4.5 (2.4–5.3) a	67.0 (52.0–70.3)	27.0 (25.0–30.0)	4.7 (3.6–6.5)	7.3 (6.3–11.0)	0.6 (0.3–0.7)
	**SF**	384 (374–402)	9.4 (7.3–12.0)	3.9 (3.0–5.8) a	67.6 (65.0–70.4)	26.2 (17.5–27.7)	5.5 (4.9–5.9)	6.3 (5.6–7.0)	0.5 (0.4–0.7)
	**HF**	419 (411–442)	17.0 (13.5–20.0)	3.5 (2.3–4.3) b	55.5 (49.0–56.5)	19.0 (17.5–22.0)	12.5 (7.9–14.0)	9.0 (8.0–9.7)	0.3 (0.3–0.4)
**Flakes**	**NO**	377 (371–385) a	1.5 (1.0–2.6) b	0.4 (0.3–0.7) b	81.0 (76.0–82.0) a	8.9 (6.1–18.5)	4.0 (3.0–5.2) b	8.0 (7.3–9.4) b	1.0 (0.5–1.6) a
	**SF**	379 (373–385) a	2.0 (1.5–3.0) b	0.4 (0.3–1.3) a,b	79.0 (76.3–81.0) a	15.0 (7.8–17.7)	4.5 (3.8–5.5) b	8.1 (7.3–9.2) b	0.8 (0.3–1.0) a
	**HF**	370 (360–390) b	6.6 (2.5–7.0) a	1.2 (0.5–1.5) a	63.0 (58.6–68.0) b	7.7 (1.4–16.0)	9.9 (8.4–11.6) a	12.0 (9.3–12.9) a	0.3 (0.0–0.8) b
**Bran cereals**	**NO**	330 (311–371)	4.0 (2.7–7.0)	0.7 (0.5–2.0)	54.0 (40.0–58.0)	2.0 (2.0–3.0)	21.0 (12.0–27.5)	13.0 (12.0–13.0)	0.1 (0.0–0.1)
	**SF**	/	/	/	/	/	/	/	/
	**HF**	321 (309–344)	4.3 (3.5–7.3)	0.9 (0.7–1.2)	40.7 (35.0–48.0)	5.9 (1.3–17.0)	31.3 (16.0–35.0)	14.5 (13.0–16.0)	0.4 (0.0–1.3)
**Puffed cereals**	**NO**	382 (376–397)	2.8 (1.8–4.0)	0.6 (0.4–1.0)	79.0 (75.9–85.0)	11.5 (0.8–27.0)	3.0 (2.3–5.5) b	7.1 (6.3–9.9)	0.0 (0.0–0.6)
	**SF**	377 (364–400)	3.3 (3.1–5.1)	0.5 (0.5–0.6)	77.0 (68.0–79.0)	41.0 (0.6–46.0)	5.5 (5.0–9.4) a,b	7.5 (7.0–11.5)	0.0 (0.0–0.0)
	**HF**	367 (365–374)	3.1 (2.9–5.0)	0.6 (0.5–0.7)	68.0 (66.0–75.9)	1.5 (0.6–15.0)	8.5 (7.0–9.4) a	11.0 (6.9–11.5)	0.0 (0.0–0.0)
**Others**	**NO**	390 (385–438) a,b	3.8 (2.8–12.7) b	1.6 (0.9–3.0)	77.5 (70.8–80.8) a	28.7 (25.0–30.0) a	3.8 (2.9–5.5) b	7.4 (6.4–8.0) b	0.7 (0.5–0.9) a
	**SF**	390 (379–394) b	3.6 (2.7–5.5) b	1.3 (0.8–2.3)	78.0 (73.7–78.5) a	23.5 (21.7–25.5) a,b	5.2 (4.9–5.9) b	8.2 (7.2–8.4) b	0.7 (0.4–0.8) a,b
	**HF**	428 (395–449) a	15.0 (7.4–18.0) a	2.2 (1.4–3.4)	63.0 (59.0–68.7) b	21.0 (19.0–22.9) b	7.8 (7.2–8.4) a	9.1 (8.7–10.0) a	0.4 (0.2–0.6) b

For each category, different lowercase letters in the same column indicate significant differences among types (Kruskal–Wallis test for independent samples with multiple pairwise comparisons or Mann-Whitney test for independent samples for bran cereal products, $p < 0.05$). Legend: HF, High in fiber; NO, No fiber-related claim; SF, Source of fiber; SFA, saturates.
