# Supplementary material for: Breakfast Cereals Carrying Fibre-Related Claims: Do They Have a Better Nutritional Composition Than Those without Such Claims? Results from the Food Labelling of Italian Products (FLIP) Study
Source: Foods. 2021 Sep 19;10(9):2225. doi: 10.3390/foods10092225 (PMC8467444; doi:10.3390/foods10092225)
Supplement: Supplementary file 1 [file foods-10-02225-s001.zip › Suppl Table 2.pdf]

Supplementary Table S2. Energy, macronutrients, and salt content across breakfast cereals organised according to the absence or presence of a fibre-related nutrition claim by considering the presence or absence of other nutrition claims, health claims or other declarations on the food pack.

	Claim		Energy (kcal/100 g)	Total fat (g/100 g)	SFA (g/100 g)	Total carbohydrates (g/100 g)	Sugars (g/100 g)	Fibre (g/100 g)	Protein (g/100 g)	Salt (g/100 g)
Without NC on fibre	NC on fat	No	388 (375–422)*	4.2 (1.8–12.4)*	1.4 (0.4–3.9)*	74.0 (65.0–81.0)	22.0 (8.2–28.3)*	4.5 (3.0–6.6)	8.0 (7.0–9.4)	0.6 (0.2–1.0)
		Yes	378 (371–384)	1.5 (1.1–2.1)	0.4 (0.3–0.5)	81.0 (80.0–83.0)*	11.0 (6.0–16.1)	3.3 (2.8–5.9)	7.9 (7.1–8.0)	0.8 (0.4–1.1)
	NC on salt	No	385 (375–408)	3.8 (1.6–10.3)	1.1 (0.4–3.5)	76.0 (66.0–81.4)	21.0 (7.8–28.0)*	4.5 (3.0–6.5)	8.0 (7.0–9.3)	0.6 (0.2–1.0)
		Yes	376 (376–376)	2.5 (2.5–2.5)	0.5 (0.5–0.5)	78.0 (78.0–78.0)	0.1 (0.1–0.1)	2.5 (2.5–2.5)	9.0 (9.0–9.0)	0.0 (0.0–0.0)
	NC on vitamins and minerals	No	385 (373–425)	4.0 (1.6–12.4)	1.1 (0.4–3.2)	75.9 (64.0–81.0)	20.0 (6.0–27.0)	4.8 (3.0–6.7)	8.0 (7.1–9.7)*	0.5 (0.1–1.0)
		Yes	387 (376–401)	3.3 (1.6–9.5)	1.0 (0.4–3.7)	76.5 (69.0–81.5)	23.6 (13.0–28.1)*	4.1 (3.0–5.8)	7.4 (6.9–9.0)	0.8 (0.5–1.0)*
	NC on sugar	No	386 (375–415)*	3.8 (1.6–11.0)*	1.2 (0.4–3.8)*	76.0 (66.0–81.4)	22.0 (8.2–28.0)*	4.4 (3.0–6.4)	8.0 (7.0–9.3)	0.6 (0.2–1.0)*
		Yes	371 (349–385)	1.0 (1.0–2.5)	0.3 (0.2–0.3)	80.0 (64.0–84.0)	1.1 (1.0–4.8)	5.0 (3.4–10.0)	8.0 (8.0–13.3)	0.0 (0.0–0.4)
	NC on protein	No	385 (375–408)	3.8 (1.6–10.3)	1.1 (0.4–3.5)	76.0 (66.0–81.4)	21.0 (7.7–28.0)	4.5 (3.0–6.5)	8.0 (7.0–9.3)	0.6 (0.2–1.0)
		Yes	/	/	/	/	/	/	/	/
	HC	No	386 (375–416)	3.8 (1.6–12.0)	1.1 (0.4–3.8)	77.0 (67.0–81.1)	21.0 (6.6–28.0)	4.4 (3.0–6.2)	8.0 (7.0–9.2)	0.6 (0.2–1.0)
		Yes	385 (374–396)	2.7 (1.8–8.9)	0.9 (0.5–2.6)	74.4 (64.0–82.0)	20.0 (10.9–27.5)	6.1 (3.5–9.4)*	9.1 (6.9–9.9)	0.8 (0.6–1.0)
	Organic	No	389 (376–444)*	4.3 (1.8–14.0)*	1.6 (0.5–4.2)*	74.8 (65.0–81.5)	23.0 (17.0–28.7)*	4.4 (3.0–6.3)	7.7 (6.9–9.2)	0.7 (0.4–1.0)*
		Yes	380 (371–397)	2.6 (1.1–5.1)	0.5 (0.3–1.3)	78.0 (68.0–81.0)	6.1 (1.1–20.0)	4.7 (3.0–7.0)	8.0 (7.3–10.0)*	0.0 (0.0–1.0)
	Branded	No	385 (376–407)	3.6 (1.5–9.6)	1.0 (0.4–3.6)	78.0 (69.0–82.0)*	21.5 (7.4–27.0)	4.0 (2.9–5.8)	7.9 (7.0–9.1)	0.6 (0.3–1.0)
		Yes	387 (372–408)	4.0 (2.1–14.0)	1.3 (0.4–3.2)	72.9 (62.2–80.0)	20.0 (7.7–28.3)	20.0 (7.7–28.3)*	8.0 (7.0–10.8)	0.7 (0.1–1.0)

	**Gluten-free**	**No**	385 (375–408)	3.8 (1.6–10.2)	1.1 (0.4–3.8)	76.0 (66.5–81.4)	21.5 (8.0–28.0)	21.5 (8.0–28.0)	8.0 (7.0–9.3)	0.6 (0.2–1.0)
		**Yes**	387 (371–400)	2.0 (1.0–18.0)	0.5 (0.2–2.1)	80.5 (52.0–81.0)	8.2 (4.8–9.1)	8.2 (4.8–9.1)	7.8 (7.3–11.0)	0.7 (0.1–1.9)
**Source of fibre**	**NC on fat**	**No**	385 (375–400)	5.8 (2.9–9.8)*	2.5 (0.7–3.8)*	72.9 (66.8–78.0)	21.8 (16.0–26.6)*	5.4 (4.8–6.0)*	7.5 (6.5–8.4)	0.5 (0.3–0.8)
		**Yes**	378 (373–380)	1.7 (1.5–2.0)	0.4 (0.3–0.4)	81.0 (77.0–81.0)*	9.0 (6.0–11.0)	4.0 (3.5–4.7)	8.0 (7.0–10.0)	1.0 (0.3–1.0)
	**NC on salt**	**No**	382 (375–394)	4.3 (2.0–8.8)	1.6 (0.5–3.5)	76.0 (67.5–79.0)*	19.0 (110–25.7)	5.1 (4.2–5.8)	7.5 (6.7–8.7)	0.6 (0.4–0.8)*
		**Yes**	413 (382–440)	13.6 (9.7–17.1)*	2.0 (1.7–2.8)	63.7 (57.2–69.7)	27.1 (15.4–33.0)	5.5 (5.0–8.6)	7.6 (5.3–11.8)	0.1 (0.0–0.2)
	**NC on vitamins and minerals**	**No**	379 (372–394)	5.1 (2.5–10.1)	1.6 (0.5–3.5)	74.7 (67.0–78.0)	19.0 (6.0–26.6)	5.0 (4.5–5.5)	7.5 (6.5–9.0)	0.4 (0.2–0.8)
		**Yes**	383 (375–397)	4.7 (1.7–7.3)	2.0 (0.5–3.3)	76.1 (68.3–79.0)	21.8 (15.3–25.7)	5.4 (4.2–5.9)	7.6 (6.8–8.6)	0.7 (0.3–0.8)
	**NC on sugar**	**No**	382 (375–399)	4.3 (2.0–8.0)	1.6 (0.5–3.3)	76.0 (67.5–79.0)	21.0 (15.0–26.4)*	5.2 (4.2–5.8)	7.7 (6.5–8.9)	0.6 (0.3–0.8)
		**Yes**	349 (337–385)	10.0 (9.6–11.5)	4.9 (2.2–6.1)	67.0 (66.5–69.7)	1.9 (1.5–7.6)	5.3 (4.8–5.5)	7.4 (6.8–8.0)	0.4 (0.0–0.5)
	**NC on protein**	**No**	382 (374–394)	4.4 (2.0–9.0)	1.6 (0.5–3.5)	75.1 (67.1–79.0)	20.7 (9.5–26.0)*	5.1 (4.2–5.8)	7.5 (6.7–8.7)	0.5 (0.3–0.8)
		**Yes**	425 (423–426)	15.5 (15.0–16.0)	3.8 (2.5–5.1)	50.0 (44.0–56.0)	17.5 (16.0–19.0)	6.4 (5.6–7.1)	18.5 (14.0–23.0)*	0.5 (0.4–0.6)
	**HC**	**No**	382 (374–400)	5.0 (2.5–9.7)	1.6 (0.6–3.4)	75.6 (67.1–79.0)	20.9 (8.2–26.2)	5.0 (4.2–5.5)	7.6 (6.8–9.0)	0.4 (0.2–0.8)
		**Yes**	382 (375–393)	4.4 (1.7–7.4)	2.5 (0.4–3.6)	73.8 (65.6–79.0)	19.0 (15.0–24.9)	5.6 (5.1–6.9)*	7.5 (6.7–8.7)	0.7 (0.5–0.8)
	**Organic**	**No**	382 (374–399)	5.6 (2.5–9.6)	2.4 (0.6–3.6)*	74.7 (66.8–79.0)	21.0 (15.0–26.0)	5.3 (4.2–6.0)	7.4 (6.7–8.4)	0.5 (0.3–0.8)
		**Yes**	380 (378–393)	3.1 (2.0–4.2)	0.6 (0.4–1.0)	77.5 (73.1–79.5)	13.9 (4.8–31.6)	5.0 (4.4–5.3)	8.2 (7.3–9.5)	0.5 (0.0–1.0)
	**Branded**	**No**	383 (376–397)	3.1 (1.7–5.4)	0.8 (0.4–2.7)	78.5 (75.0–81.0)*	17.4 (10.3–24.4)	4.5 (3.9–5.6)	8.1 (7.1–9.1)	0.4 (0.3–0.8)
		**Yes**	380 (375–394)	7.4 (3.3–12.0)*	3.0 (1.6–3.9)*	69.3 (65.0–75.1)	22.3 (15.0–26.4)	5.5 (5.0–6.0)*	7.1 (6.2–8.4)	0.6 (0.4–0.8)
	**Gluten free**	**No**	382 (374–394)	5.0 (2.3–9.0)	1.7 (0.5–3.5)	75.1 (67.0–79.0)	19.0 (11.0–26.0)	5.4 (4.5–6.0)	7.6 (6.7–9.0)*	0.5 (0.3–0.8)
		**Yes**	382 (378–434)	2.5 (2.0–15.0)	1.6 (0.6–5.0)	78.0 (68.0–81.0)	21.0 (8.5–26.6)	4.2 (3.2–4.9)	7.3 (5.9–8.4)	0.8 (0.2–0.8)

**High in fibre**	**NC on fat**	**No**	377 (360–437)	7.5 (4.0–15.0)	1.5 (0.8–3.1)*	60.3 (56.0–65.0)	17.0 (8.0–22.0)	9.3 (7.4–14.0)	10.0 (8.5–13.0)	0.3 (0.1–0.6)
		**Yes**	367 (358–393)	7.0 (2.5–7.4)	0.9 (0.5–1.2)	67.0 (61.0–68.7)	7.0 (1.5–18.0)	8.7 (8.1–10.2)	11.5 (9.2–13.0)	0.2 (0.1–0.6)
	**NC on salt**	**No**	378 (360–431)	7.3 (3.9–15.0)	1.3 (0.8–2.6)	61.0 (56.8–65.7)	17.8 (8.5–22.0)	9.0 (7.4–14.0)	10.0 (8.6–12.0)	0.3 (0.1–0.6)*
		**Yes**	365 (352–437)	7.7 (4.5–15.0)	1.3 (0.8–3.4)	58.0 (49.0–62.9)	2.0 (1.1–18.0)	9.3 (8.0–16.0)	13.0 (9.5–16.0)	0.0 (0.0–0.1)
	**NC on vitamins and minerals**	**No**	376 (360–437)	7.3 (4.4–15.0)	1.4 (0.9–3.1)	61.0 (57.0–65.0)	17.0 (3.8–21.0)	8.5 (7.2–11.0)	10.1 (8.5–12.5)	0.2 (0.1–0.5)
		**Yes**	379 (352–419)	7.2 (3.0–15.0)	1.2 (0.7–2.1)	58.0 (49.0–66.0)	17.5 (12.0–21.0)	12.5 (8.5–16.0)*	9.5 (8.7–14.0)	0.4 (0.2–0.9)*
	**NC on sugar**	**No**	380 (360–435)	7.4 (3.9–15.0)	1.3 (0.8–3.1)	61.0 (56.0–65.7)	18.0 (8.8–22.0)*	8.8 (7.4–14.0)	10.0 (8.7–13.0)	0.3 (0.1–0.6)
		**Yes**	363 (344–431)	7.0 (4.5–15.0)	1.3 (0.8–1.9)	57.4 (40.3–65.0)	2.5 (1.3–12.0)	11.0 (8.5–16.0)	11.8 (8.6–13.0)	0.2 (0.0–0.8)
	**NC on protein**	**No**	380 (360–436)*	7.4 (3.9–15.0)*	1.4 (0.8–2.9)*	61.0 (56.7–65.9)	17.8 (8.3–21.5)*	8.8 (7.5–14.0)	10.0 (8.6–12.7)	0.3 (0.1–0.6)*
		**Yes**	360 (357–365)	7.0 (4.5–7.0)	1.2 (1.1–1.3)	51.0 (49.4–57.0)	1.3 (1.2–1.7)	10.4 (9.5–14.0)	14.8 (12.5–16.0)*	0.1 (0.0–0.2)
	**HC**	**No**	378 (360–437)	7.4 (3.9–15.0)	1.3 (0.8–3.4)	61.5 (57.0–66.0)*	18.0 (6.4–22.0)	8.4 (7.3–10.4)	10.0 (8.5–12.7)	0.3 (0.1–0.6)
		**Yes**	374 (352–411)	7.3 (4.5–10.0)	1.2 (0.9–1.9)	58.0 (48.0–63.0)	13.0 (1.3–19.0)	14.0 (8.7–16.0)*	11.5 (9.0–14.0)	0.3 (0.1–0.6)
	**Organic**	**No**	390 (360–440)	7.9 (4.0–17.0)	1.8 (0.8–3.1)	60.0 (55.5–65.0)	18.0 (12.2–22.0)	9.5 (7.5–14.0)	9.5 (8.5–12.5)	0.3 (0.2–0.6)
		**Yes**	368 (360–396)	7.0 (3.1–8.1)*	1.1 (0.7–1.5)*	62.5 (58.0–66.5)	7.2 (1.4–18.5)	8.4 (7.4–11.3)	12.0 (9.3–13.0)	0.1 (0.0–0.2)
	**Branded**	**No**	371 (354–403)	5.0 (3.0–8.3)	1.0 (0.6–3.1)	63.0 (57.4–68.0)	18.0 (8.5–22.0)*	9.0 (7.3–15.4)	11.6 (8.7–13.0)*	0.4 (0.1–0.8)*
		**Yes**	385 (360–437)	8.5 (5.5–16.0)*	1.5 (1.0–2.6)	59.0 (56.0–65.0)	16.2 (3.6–20.4)	9.1 (7.5–13.0)	10.0 (8.6–12.0)	0.2 (0.1–0.6)
	**Gluten free**	**No**	377 (360–435)	7.3 (3.9–15.0)	1.3 (0.8–2.6)	61.0 (56.0–65.0)	17.3 (5.0–21.0)	9.0 (7.5–14.0)	10.6 (8.8–13.0)	0.3 (0.1–0.6)
		**Yes**	360 (357–390)	5.3 (1.9–8.9)	0.8 (0.6–3.1)	67.0 (54.0–81.0)	8.5 (6.6–15.0)	10.0 (7.1–20.0)	6.5 (6.0–7.1)	0.3 (0.1–0.3)

For each category, asterisks in the same column indicate significant differences between the values of each of the considered nutritional value in products boasting or not the claim (Mann-Whitney test for independent samples for bran cereals products, p <0.05). Legend: HC, Health claim; NC, Nutrition claim; SFA, saturates.
